# Novel Sensors and Data-Driven Approach to Support Prevention, Diagnosis, and Treatment Planning of Cerebrovascular Accidents: Protocol for a Multimodal Data Collection Study With Novel Sensors

**DOI:** 10.2196/86930

**Published:** 2026-10-08

**Authors:** Milla Immonen, Hilkka Liedes, Aysen Degerli, Ilona Ruotsalainen, Juha Pajula, Mika Hilvo, Heidi Similä, Adil Umer, Mark van Gils, Miia Jansson, Kirsi Maaria Rasmus, Minna Pikkarainen, Petri Huhtinen, Mikko Kärppä, Julius Francis Gomes, Hany Ferdinando, Miguel Bordallo López, Teemu Myllylä, Vesa Kiviniemi, Mikael von und zu Fraunberg, Pekka Jäkälä

**Affiliations:** 1 VTT Technical Research Centre of Finland Ltd Oulu Finland; 2 Future Healthcare Services Expertise Group Northern Well-being and services Lapland University of Applied Sciences Rovaniemi Finland; 3 VTT Technical Research Centre of Finland Ltd Tampere Finland; 4 VTT Technical Research Centre of Finland Ltd Espoo Finland; 5 Faculty of Medicine and Health Technology Tampere University Tampere Finland; 6 Research Unit of Health Sciences and Technology Faculty of Medicine University of Oulu Oulu, North Ostrobothnia Finland; 7 Oulu University Hospital Oulu Finland; 8 Health and Biomedical Sciences Royal Melbourne Institute of Technology Melbourne, Victoria Australia; 9 Martti Ahtisaari Institute Oulu Business School University of Oulu Oulu, North Ostrobothnia Finland; 10 Faculty of Health Sciences and Faculty of Technology, Art and Design OsloMet – Oslo Metropolitan University Oslo Norway; 11 Optomed Plc Oulu Finland; 12 Research Unit of Clinical Medicine Faculty of Medicine University of Oulu Oulu Finland; 13 Computer Science and Engineering Faculty of Information Technology and Electrical Engineering University of Oulu Oulu, North Ostrobothnia Finland; 14 Medical Research Center Oulu Oulu Finland; 15 Kuopio University Hospital Kuopio Finland; 16 Institute of Clinical Medicine, School of Medicine Faculty of Health Sciences University of Eastern Finland Kuopio Finland

**Keywords:** stroke, cerebrovascular diseases, data analysis, transient ischemic attack, sensors

## Abstract

**Background:**

Stroke is the second leading cause of death worldwide. Cerebrovascular diseases (CVDs), including strokes and transient ischemic attacks (TIAs), cause long-term disabilities and economic burdens. Strokes can be ischemic, caused by blood clots; or hemorrhagic, caused by bleeding in the brain. Immediate diagnosis and treatment are crucial. Proper management of TIAs is essential due to the high risk of subsequent strokes. Currently, wearable sensors, AI-based prediction, and automatic video analysis are not used in CVD diagnosis and recovery estimation.

**Objective:**

This paper describes the design, cohort characteristics, and multimodal dataset of the Stroke-Data study conducted at 2 university hospitals in Finland. Although findings from individual components of the study have been reported elsewhere, this paper provides the first comprehensive description of the overall study design, cohort, and multimodal data collection protocol. The study collected a unique multimodal dataset comprising sensor data, video recordings, clinical assessments, questionnaires, and health record data from healthy controls and patients with stroke and TIA to support the development of AI, sensor-based, and video-based methods for CVD diagnosis and recovery estimation.

**Methods:**

Stroke-Data was a prospective national multicenter study conducted at Oulu University Hospital and Kuopio University Hospital from October 2021 to December 2022. A rich multimodal dataset from patients with stroke and TIA and healthy controls was collected by study nurses and a research assistant. Inclusion criteria for patients were age >18 years and a diagnosis of cerebrovascular accident or TIA within the previous 3 days. Healthy control participants were aged 18 years and older and had no major chronic diseases, although blood pressure and cholesterol medications were permitted. Data collection included electroencephalogram; electrocardiogram; near-infrared spectroscopy; accelerometers to analyze gait and balance; retinal fundus images; video recordings of neurological examinations to analyze the face, body movements, and speech; clinical assessments; questionnaires; and electronic health records.

**Results:**

In total, 263 participants were recruited, of whom 123 were controls (median age 59, IQR 45-72 years; female: 84/123, 68.3%), 31 were patients with TIA (median age 71, IQR 60-80 years; female: 8/31, 25.8%), and 103 were patients with stroke (median age 70, IQR 59-76 years; female: 42/102, 41.2%, sex data missing from 1 participant). A total of 6 participants were excluded (5 had diagnoses other than TIA or stroke and 1 control participant had a previous CVD).

**Conclusions:**

We successfully conducted a multicenter multimodal data collection study on healthy controls and patients with stroke and TIA. This protocol paper provides a comprehensive methodological description of the Stroke-data study, serving as a reference for previously published and future analyses based on the dataset. The protocol is unique in combining physiological sensor data, video recordings, clinical assessments, questionnaires, and health record data from patients with stroke and TIA and healthy controls in a multicenter setting.

**International Registered Report Identifier (IRRID):**

DERR1-10.2196/86930

## Introduction

In Finland, 1.5% of the national population encounter stroke [1]. Stroke also has a major global impact, where 12.2 million stroke incidents occurred in 2019 worldwide, and ischemic stroke constituted 62.4% (7.6 million) of all new strokes [2]. Indeed, based on the data in 2019, globally, stroke is the second leading cause of death and the third leading cause of combined death and disability. This is because cerebrovascular disorders cause permanent symptoms in approximately half of the survivors and decrease the amount of quality-adjusted life years. These permanent symptoms lead to reduced capacity for work and increased costs for societies. Thus, it is crucial to diagnose and treat cerebrovascular diseases (CVDs) correctly and without delay to optimize rehabilitation.

CVDs include strokes and transient ischemic attacks (TIAs). A stroke can be either an ischemic stroke or a hemorrhagic stroke. An ischemic stroke is caused by a blood clot blocking an artery to the brain (thrombotic stroke) or by a piece of plaque or thrombus blocking an artery (embolic stroke). Hemorrhagic stroke is caused by bleeding into the brain by the rupture of a blood vessel [3]. Hemorrhagic stroke can be either intracerebral hemorrhage, where the bleeding occurs into the brain parenchyma; or subarachnoid hemorrhage, where the bleeding occurs into the subarachnoid space. TIA is a temporary accident or disorder resembling stroke, but the symptoms are transient and do not leave permanent damage [4]. However, it is also important to treat patients with TIA properly, as they have an increased risk of having a stroke later; it is estimated that 10%-20% of patients with their first TIA may experience a stroke within 90 days [5,6].

Immediate diagnostics and treatment are very important in CVDs. The assessment and diagnosis of stroke can be performed with the help of imaging techniques, computed tomography (CT) or magnetic resonance imaging, and physical and neurological examination tests (eg, National Institutes of Health Stroke Scale [NIHSS]), in addition to questionnaire data collected from the patients [3]. For the treatment decision, a CT scan is typically made immediately upon arrival at the hospital [7]. In addition, the doctor needs comprehensive information, such as onset of symptoms, previous diseases, medication, and information on risk factors. For the differential diagnostics of CVDs, the clinical scales including Face, Arms, Speech, Time [8], Cincinnati Prehospital Stroke Scale [9], and Finnish Prehospital Stroke Scale [10,11] are used. Ischemic stroke can be treated with thrombolytic therapy in some patients within a 4.5-hour time window calculated from the onset of symptoms and in carefully selected patients within a 9-hour time window if perfusion imaging shows salvageable tissue [12]. Furthermore, ischemic stroke caused by proximal brain artery occlusion can be treated with mechanical thrombectomy within a 24-hour time window also in carefully selected patients [13]. Hemorrhagic stroke also requires urgent diagnosis and treatment, focusing on limiting hematoma expansion, controlling intracranial pressure, and managing complications, with neurosurgical intervention required in selected patients [3].

The aim of this work was to describe in detail the study design and cohort characteristics of the Stroke-Data study. Stroke-Data aimed (1) to investigate what kind of new sensors and data-driven methods can support diagnosis of CVDs, (2) to develop models for predicting treatment response and recovery after a CVD accident, and (3) to study how the sensors and data-driven methods can support identification of new CVD risk factors and biomarkers associated with CVDs. In addition, the collected multimodal data enable the development of data-driven models for classification and prediction tasks, such as differentiating between stroke, TIA, and healthy controls. The aim was to collect data from healthy controls and patients with TIA or stroke using electroencephalography (EEG); electrocardiography (ECG); near-infrared spectroscopy (NIRS); accelerometers to analyze gait and balance; retinal fundus images; video recordings of neurological examination to analyze the face, body movements, and speech; clinical assessments; questionnaires; and electronic health records (EHRs). While this study is not designed for prospective prediction of first-ever stroke or TIA, the collected data may inform future studies focusing on primary prevention.

## Methods

### Study Setting

The Stroke-Data collection was performed as a prospective national multicenter study at 2 hospitals in Finland: Oulu University Hospital (OUH) and Kuopio University Hospital (KUH), during the period between October 2021 and December 2022. The data collection was performed by 2 study nurses in both locations and assisted by a research assistant in Oulu. The study was based on direct observations, and the aim was to collect information from healthy control participants and patients who have had a stroke or TIA. Before the actual data collection started, the nurses rehearsed the study protocol and use of the data collection devices. In addition, the personnel in both hospitals were informed about the study.

### Study Participants: Inclusion and Exclusion Criteria

To be included in the main study group, the patient had to be admitted to a neurological or neurosurgical ward at the hospital, be 18 years and older, and been diagnosed with a cerebrovascular accident or TIA within the last 0-3 days according to the International Classification of Diseases (ICD-10: I60-69 and G45; ICD-9: 430-438; and ICD-8: 430-438). Healthy people were included in the control group, if they were 18 years and older and had no history of CVD or other major chronic diseases expected to affect physiological or sensor-based measurements. The use of blood pressure and cholesterol medications was permitted. People who had other long-term medication, smoked, or had other addictions were excluded from the study. This was done to establish a relatively homogeneous control group without major behavioral or vascular risk factors that could influence physiological and sensor-based measurements and act as confounding factors in multimodal signal analysis. Prisoners or forensic patients, patients with reduced capabilities, and pregnant or nursing women were excluded from all study groups.

The stroke and TIA classifications were based on the final clinical diagnosis established during the participants’ hospital stay, in accordance with the Finnish Current Care Guidelines. Stroke diagnoses were confirmed using clinical assessment and neuroimaging findings. Participants whose final diagnosis was revised to a condition other than stroke or TIA were excluded from the final cohort (n=5). Healthy controls were recruited according to the predefined eligibility criteria and screened by the study nurse using a questionnaire covering medical history, chronic diseases, medication use, smoking status, previous cerebrovascular events, and other relevant health conditions. One control participant was excluded from the final cohort because a previous CVD was identified. All analyses were performed using these final classifications.

### Recruitment

The patients for the study were recruited at OUH and KUH by the study nurses with the assistance of health care professionals, immediately after the patients had been diagnosed with a cerebrovascular accident or TIA, upon arrival to the hospital, and after they had received the most acute treatment and were treated in the ward at the time of recruitment. The recruitment happened within the first 3 days after hospital admission.

The healthy controls in Oulu were recruited by the study nurse from the OUH staff email distribution list, whereas in Kuopio, they were recruited by the study nurses, with the help of newsletter and wall announcements, from service centers for older people, community colleges, libraries, the Alzheimer Society of Finland, and pensioners’ associations. The recruitment of healthy controls aimed to achieve a similar age and sex distribution to that of patient groups; however, this was not fully achieved due to recruitment challenges.

### Ethical Considerations

The study was conducted according to the principles of the Declaration of Helsinki and approved by the ethics committee of North Ostrobothnia Hospital District (22/2021; current name: the regional medical research ethics committee of the Wellbeing Services County of North Ostrobothnia). The study was registered and approved as a clinical investigation of medical devices by the Finnish Medicines Agency Fimea (FIMEA/2021/003738). In addition, the trial was approved by the administration of OUH and KUH. Written informed voluntary consent was obtained at the time of recruitment. The study protocol was explained to the participants. The participants were given a written description of their rights, the purpose and nature of the research, the methods used in it, and possible risks and harm (Act on Medical Research 488/1999). Participants had the right to withdraw from the study without a reason at any stage without affecting their care, treatment, or services in any way. Two copies of the consent forms were signed, one of which remained with the patient him- or herself, and the other was kept in a prearranged lock cabinet on the hospital premises by the study nurse. A personal study ID was used for all participants to pseudonymize the data.

### Data Collection Protocol

### Overview

The data collection protocol included data collection from the patient data register, clinical assessments and questionnaires, and data collection with devices. Follow-up telephone interviews were conducted 3 months after hospital discharge and included the assessment of the modified Rankin Scale (mRS), patient-reported outcome measures, and patient-reported experience measures. The data collection with the devices, assessments, and questionnaires was similar for all the study groups. However, for healthy controls, neither the patient register data were collected nor the follow-up telephone interviews made. The patients were recruited between October 2021 and September 2022, and the healthy controls between October 2021 and December 2022. The data collection, including the follow-up telephone interviews, was performed between October 2021 and December 2022. The duration of individual recordings varied between participants because data collection was performed according to a standardized protocol but adapted to participants’ clinical condition and the time required to complete the assessment tasks.

### Patients With Stroke and TIA

After receiving the most acute treatment, patients with stroke and TIA were enrolled in the hospital ward after discussing with health care professionals, and data collection was started either on the same or the following day. The data collection took altogether 1-2 hours per participant, but if needed, the data collection was split into 2 or 3 separate sessions during the same day or 2 consecutive days. The health condition of patients was considered while collecting the data, and if the health condition worsened, the data collection was either cancelled or postponed. All assessments were conducted within 3 days of hospital admission. Exact symptom-onset times, ambulance-call times, and treatment-to-assessment intervals were not systematically available for the entire cohort. Therefore, detailed intervals between symptom onset, hospital admission, acute treatment, and individual measurements were not analyzed. The data collection protocol is illustrated in Figure 1.

**Figure 1:**
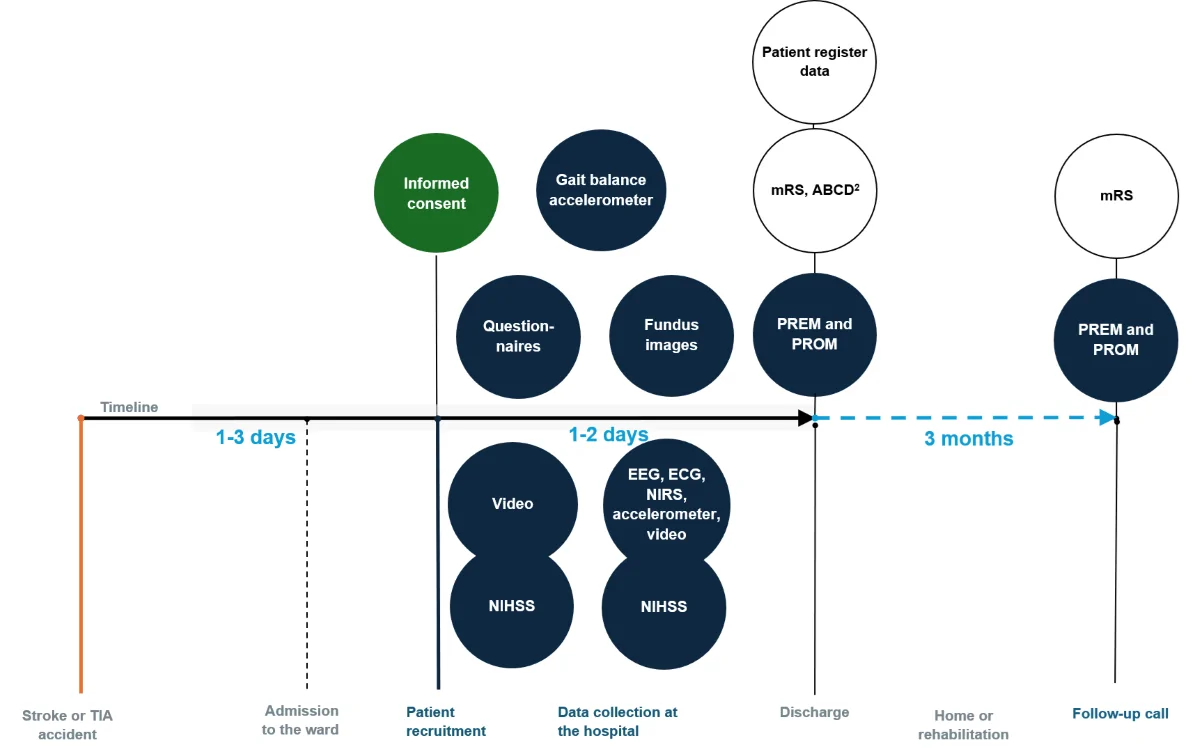
Data collection protocol for patients with stroke and TIA in a multicenter study conducted at 2 Finnish university hospitals between October 2021 and December 2022. Intervals on the horizontal time axis are not according to scale. ABCD2: a risk stratification score based on age, blood pressure, clinical features, duration, and diabetes; ECG: electrocardiography; EEG: electroencephalography; mRS: modified Rankin Scale; NIHSS: National Institutes of Health Stroke Scale; NIRS: near-infrared spectroscopy; PREM: patient-reported experience measure; PROM: patient-reported outcome measure; TIA: transient ischemic attack.

### Healthy Controls

The data collection of the healthy control participants was performed during 1 session at an agreed time slot. The health condition of healthy control participants was considered while collecting the data, and if their health condition declined, the data collection was either cancelled or postponed. The data collection protocol is illustrated in Figure 2.

**Figure 2:**
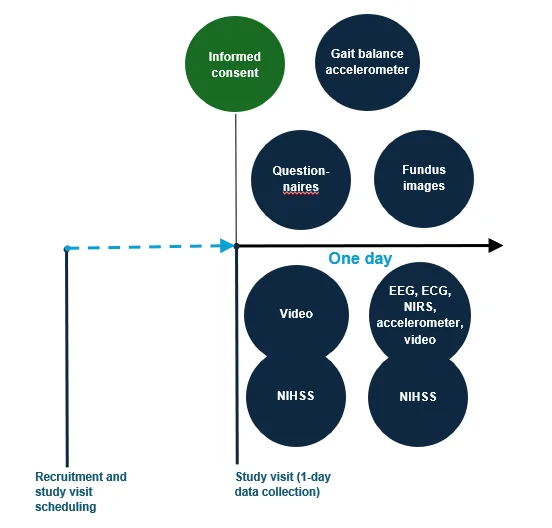
Data collection protocol for healthy control participants in a prospective multicenter study conducted at Oulu University Hospital and Kuopio University Hospital, Finland, between October 2021 and December 2022. ECG: electrocardiography; EEG: electroencephalography; NIHSS: National Institutes of Health Stroke Scale; NIRS: near-infrared spectroscopy.

### Selected Technologies

#### EEG and ECG

EEG was used to record brain activity during standardized clinical assessment tasks. EEG and quantitative EEG have previously been investigated as potential sources of stroke-related biomarkers [14], including interhemispheric correlation coefficients [15]. In this study, EEG recordings were acquired using a Conformité Européenne (European Conformity)–marked Bittium NeurOne system (Bittium Biosignals Ltd). Data were collected using a 32-electrode headcap and 1 additional ECG electrode connected to the NeurOne jackbox.

#### NIRS and Accelerometer for Detection of Head Motion

Functional near-infrared spectroscopy (fNIRS) techniques in brain monitoring typically use the spectral range of 650 to 950 nm, where light attenuation in tissue is low enough to enable reaching the cerebral cortex of the brain. However, it is important to note that NIRS can only provide information from the brain cortex, up to a depth of approximately 2 cm [16]. In general, NIRS can detect intracranial hematomas in the brain cortex by exploiting the fact that extravascular blood absorbs near-infrared light more than intravascular blood. This phenomenon occurs due to the higher hemoglobin concentration present in acute hematoma compared to the levels found in regular brain tissue, where blood is confined within vessels.

fNIRS has already been used in earlier studies concerning stroke diagnostics. A systematic review of fNIRS for stroke found several applications related to motor recovery, cortical function recovery, monitoring hemodynamic changes and cerebral blood oxygenation, evaluating the risk for perioperative stroke, and in therapeutic tools, especially, for TIA [17]. Another study found that patients with stroke had more variability in head motions in the x axis and less in the z axis, where findings increased with growing severity [18]. This finding equates to more movement in tilting the head from left to right (as in, “tilting ear to shoulder”), and less movement in shaking the head from left to right (as in, “shaking your head no”). These may indicate movement differences due to motor system impairments or attentional differences due to contralateral neglect. Because of these potential findings, we used fNIRS and an accelerometer combined with EEG in the brain data collection, aiming to detect cerebrovascular disorders.

#### Gait

Most patients with stroke experience deficits in their walking ability that often persist in the chronic phase [19]. However, the effects of TIA on walking performance remain less well identified. Overall, walking ability has been shown to predict recovery from stroke [20], but more detailed insights from walking performance could enhance the accuracy of recovery prediction. For the gait evaluation, the participants who were able to walk 10 m independently without a walking aid were asked to walk a 10-m long walkway at their habitual walking speed. A total of 224 participants completed the walking task; however, gait data were not available for 2 control participants and 1 patient with stroke due to either a malfunctioning accelerometer or bad data quality. Thus, gait data were available for 220 participants (121 control, 70 stroke, and 29 TIA). Gait properties were measured using a tri-axial MoveSense accelerometer (36.6 mm×10.6 mm, weight 10 g) that was attached to participants’ lower back with an elastic belt and with sampling frequency of 104 Hz (range ±16 g). Using a custom-made application, the study nurse started and ended the recording at the beginning of the walking task and at the end of the task (10-m line). The recorded data consisted of raw tri-axial acceleration signals, which will be used to derive various gait-related characteristics, such as temporal, spatial, variability, asymmetry, and stability measures. The feasibility of MoveSense to collect slow and normal gait has been shown in adults [21]. The data collection protocol is described in more detail in the “Accelerometer data collection from gait” section in Multimedia Appendix 1.

#### Fundus Imaging

The known markers of elevated stroke risk in retina include venular widening, decreased fractal dimension, and increased tortuosity [22]. The objective of the fundus image collection was to analyze fundus images to reveal any signs of stroke or TIA using image processing and computer vision algorithms as proposed in our study [23]. For the data collection, the Optomed Aurora IQ Camera was used to take the fundus images of the retinas in both eyes of the participants. Accordingly, 2 fundus images from each participant’s eye were taken for analysis. The first view of the fundus image was taken to visualize the central visual field, whereas the second view showed the papilla in the middle. A total of 263 participants participated in the study, and images from 38 participants were missing due to difficulties in imaging or low quality of the collected data. Thus, the collection of fundus image data included 121 healthy controls, as well as 73 patients with stroke and 26 patients with TIA with a total of 802 fundus images. The data collection process varied in some cases. If data quality was not acceptable for the analysis, multiple images were collected from the same eye of the same view. In some cases, only 1 image was available for analysis since the collection of data was stopped due to the conditions of the participants. For more details on data collection, the protocol is described in detail in the “Fundus images” section in Multimedia Appendix 1.

#### Video

The video data collection was designed to minimize human error and variability, providing a robust dataset for subsequent analysis, by focusing on specific, observable NIHSS tasks, and using a uniform recording setup. Video analysis in stroke assessment aims to systematically identify and quantify observable symptoms indicative of stroke or TIA. The method proposed in our study [24] using the collected data was chosen to supplement the NIHSS assessment, especially in a situation of scarcity of neurologists [25]. It provides a consistent and objective measure of symptoms, particularly those that can be visually discerned, such as facial drooping or limb movement. Our motivation aligns with previous research [26,27], which emphasizes the importance of computer-assisted diagnostics in identifying alterations in facial expression, such as facial paralysis, a key stroke indicator. Recent studies have also demonstrated the potential of combining different visual data to achieve high diagnostic performance [28].

For data collection purposes, a custom-made smartphone app focusing on data security was used to record patients performing specific NIHSS tasks in the stroke unit of a hospital. The app guided study nurses through the standardized protocol, capturing 4 NIHSS tasks: facial palsy, best gaze, best language, and motor arm, using a smartphone mounted on a tripod and equipped with a circular light to ensure consistent lighting and quality. In our study, we used a selective approach to NIHSS, focusing on components that could be effectively captured and analyzed through video. Certain aspects of NIHSS were omitted due to their reliance on direct physical examination or other modalities not suited to video analysis. Each participant was recorded performing the NIHSS tasks, with each task captured in a separate video segment to facilitate detailed analysis. This methodological approach underscores a commitment to achieving a high standard of data consistency and reliability.

Details of the protocol can be found in the “Video and sound” section in Multimedia Appendix 1, which outlines the specific NIHSS components assessed, the rationale for the exclusion of other components, based on their reliance on direct physical examination or other modalities not suited to video analysis, and the methodology used in video data capture and analysis. The video collection involved 148 participants, including 54 healthy controls and 94 individuals diagnosed with stroke or TIA.

#### Assessments

Disability of the patients after stroke or TIA was assessed with the mRS [29,30]. The mRS consists of 7 grades, with higher values representing worse condition (0=no symptoms at all; 1=no significant disability despite symptoms: able to carry out all usual duties and activities; 2=slight disability: unable to carry out all previous activities but able to look after own affairs without assistance; 3=moderate disability: requiring some help but able to walk without assistance; 4=moderately severe disability: unable to walk without assistance and unable to attend to own bodily needs without assistance; 5=severe disability: bedridden, incontinent, and requiring constant nursing care and attention; and 6=dead). The study nurses assessed the mRS just before discharge or transfer to a rehabilitation unit and at the follow-up telephone interviews 3 months after discharge.

The risk of stroke after TIA was assessed with ABCD^2^ (age, blood pressure, clinical features, duration, and diabetes), a risk stratification score based on five factors: (1) age ≥60 years (1 point), (2) systolic blood pressure ≥140 mm Hg or diastolic blood pressure ≥90 mm Hg (1 point), (3) clinical features: unilateral weakness (2 points) or speech impairment without weakness (1 point), (4) duration of TIA ≥60 minutes (2 points) or duration of TIA 10-59 minutes (1 point), and (5) diabetes (1 point). The total score of ABCD^2^ is the sum of points obtained from the individual factors, thus, ranging from 0 to 7, higher points indicating higher risk [31]. The study nurses assessed the ABCD^2^ just before discharge.

#### Questionnaires

The questionnaires about demographics, cerebrovascular risk factors, and self-perceived balance confidence were filled in after arrival to the stroke ward. Whereas the questionnaires about health-related quality of life and patient experience were filled in just before discharge or transfer to a rehabilitation unit and at the follow-up visit 3 months after discharge.

Demographics included age, sex (male and female), marital status (married or registered partnership, cohabiting, divorced or widow, and single), education (primary education [≤International Standard Classification of Education 2], upper secondary education, bachelor or equivalent, and master, doctoral, or equivalent), and occupational status (employee, entrepreneur, retired, student, unable to work or on sick leave, and on parental leave or stay-at-home parent).

Cerebrovascular risk factors included height (cm), weight (kg), number of previous cerebrovascular accidents, number of diseases, high blood pressure (yes or no), use of blood pressure medication (yes or no), diabetes (yes or no), migraine (yes or no), high blood cholesterol (yes or no), and arrhythmia (yes or no). In addition, lifestyle-related cerebrovascular risk factors included smoking, stress or depression, physical activity, alcohol use, sleeping, and diet (see the “Questionnaire: Lifestyle-related cerebrovascular risk factors” section in Multimedia Appendix 1 for details).

Self-perceived balance confidence was measured with the Activities-Specific Balance Confidence (ABC) Scale [32]. The ABC Scale is a structured questionnaire with 16 items, in which individuals rate their confidence in performing various daily activities without losing balance or becoming unsteady. Each item is scored with 11 levels from 0%=no confidence to 100%=completely confident.

Health-related quality of life was evaluated with the 15D instrument [33], which is a self-administered questionnaire assessing 15 dimensions of health**:** mobility, vision, hearing, breathing, sleeping, eating, speech (communication), excretion, usual activities, mental function, discomfort and symptoms, depression, distress, vitality, and sexual activity. Each dimension has 5 levels of severity, from no problems to severe problems. The question about sexual activity was not asked from all patients because it was deemed too personal and sensitive for the patients.

Patient experience was evaluated with a questionnaire compiled based on 4 patient experience questionnaires: 11 scale by the Finnish Institute for Health and Welfare, the Nordic Patient Experience Questionnaire [34], the Generic Short Patient Experience Questionnaire [35], and the Picker Patient Experience Questionnaire [36]. The questionnaire included 17 statements, which are answered on a 5-point Likert scale, with answer alternatives ranging from 1=completely disagree to 5=completely agree. In addition, there was 1 open question for additional feedback. The same questionnaire has been used in a previous study [37].

#### EHRs

The study nurses collected the following information from the EHR system of the hospitals:

- Background information included arrival time to the hospital, discharge time, age, sex, living independently at home (yes or no), ability to perform daily activities, medications, and previous cerebrovascular diagnoses (ICD-10: I60-69 and G45; ICD-8 and ICD-9: 430-438).
- Diagnoses included the main diagnosis, other diagnoses, and treatment period diagnosis, each from 3 phases of the treatment path: check-in to hospital, discharge from hospital, and control visit. In addition, the diagnosis for deceased with details “yes-no” and code for reason were collected.
- Cerebrovascular symptoms included start time of the symptoms, duration of the symptoms (<10, 10-59, and ≥60 minutes), symptoms, and need for care assessed by paramedics.
- Cerebrovascular risk factors included BMI, smoking or snuff use, alcohol use, diabetes (yes or no), physical activity level, family history of cerebrovascular accidents, and blood lipid levels (low-density lipoprotein, high-density lipoprotein, cholesterol, and triglycerides).
- Vital signs included heart rate, results from electrocardiography, blood pressure, breathing rate, temperature, blood glucose level, and oxygen saturation.
- Imaging results included CT, CT angiography, perfusion, magnetic resonance imaging, carotid artery imaging, and Fazekas classification.
- Treatments included intravenous thrombolysis (yes or no and time stamp), mechanical thrombectomy (yes or no, time stamp, and success), surgical operations (yes or no, time stamp, and type), conservative treatments (type and time stamp), medications, and rehabilitation plan.
- Assessments at the hospital included mRS, NIHSS, balance, and Trial of Org 10172 in Acute Stroke Treatment classification. In addition, more detailed information was collected from the Kuopio stroke register. As these are available only for patients treated in KUH, details are not described here.

### Data Management

The collected data described in Table 1 were transferred in both hospitals to network drives accessible only by dedicated study nurses. After the data had been collected, the data were transferred in a pseudonymized format to a private cloud server at CSC ePouta [38], which is a service for researchers to analyze sensitive data. The University of Oulu IT department deployed a virtual machine to the ePouta service, and all analysis of the collected data was performed with this ePouta virtual machine.

**Table 1:** Summary of the multimodal data collection technologies, instruments, and parameters used in the Stroke-Data study conducted in Finland (2021-2022).

Method	Instruments used	Parameters or device settings
EEG^a^	Bittium NeurOne	Sampling rate 5000 Hz, 32 channels
NIRS^b^	Designed at the University of Oulu [39]	Sampling rate 800 Hz, 4 wavelengths with optical power of 4-10 mW
ECG^c^	ECG was collected with 1 additional ECG electrode AMBU ECG L-00 connected to NeurOne jackbox	Sampling rate 1000 Hz
Gait	MoveSense 9-axis IMU^d^ sensor, size 36.6 mm×10.6 mm, weight 10 g	104 Hz sample frequency
Fundus imaging	Optomed Aurora IQ (sw version 3.3.7)	Auto-focus and auto-exposure
Video	Stroke-Data smartphone app (VTT^e^ in-house)	Nokia X20 smartphone, Android, camera resolution: 64 megapixels, processor type: Qualcomm Snapdragon 480 5G, CPU^f^ speed: 2 GHz, and number of cores: 8
Assessments	Modified Rankin Scale [29,30]	N/A^g^
Assessments	ABCD^2h^ score [31]	N/A
Questionnaires	Demographics	N/A
Questionnaires	Cerebrovascular risk factors	N/A
Questionnaires	Activities-Specific Balance Scale [32]	N/A
Questionnaires	15D instrument [33]	N/A
Questionnaires	Patient experience	N/A
EHR^i^	Hospital systems	See details in the text

^a^EEG: electroencephalography.

^b^NIRS: near-infrared spectroscopy.

^c^ECG: electrocardiography.

^d^IMU: inertial measurement unit.

^e^VTT: Technical Research Centre of Finland Ltd.

^f^CPU: central processing unit.

^g^N/A: not applicable.

^h^ABCD^2^: a risk stratification score based on age, blood pressure, clinical features, duration, and diabetes.

^i^EHR: electronic health record.

The data access inside the virtual machine was managed with Linux user groups following the minimum access principle so that researchers had access only to that part of the data which they needed to for their specific research. Each data type had its own user group, and users were assigned to the groups according to the research permit of the study. The machine had 12 central processing unit cores, 240 GB RAM memory, and 1.5 TB storage space. Additionally, the virtual machine also had an Nvidia Tesla A100 40 GB graphics processing unit for image and video data analysis and machine learning studies.

### Sample Size

There are approximately 500-600 patients with CVD yearly in KUH, and their average stay at the hospital is 4 days. The yearly number of patients with CVD in OUH is 600-700, and their average stay at the hospital is 3.5 days. The patients who received active treatments like thrombolysis or thrombectomy come to a control visit at the ward after 3 months in both hospitals. The number of these patients is approximately 150-200 yearly per hospital. The sample included all patients matching the inclusion criteria during the study period so that the study nurses had enough time to recruit and plan the data collection for 1 patient per day. This meant that during a 6-month period it was possible to collect data from 50 to 100 patients per hospital. In addition, the aim was to collect data from 100 to 200 healthy controls. A formal statistical power calculation was not performed, as the primary aim of the study was to establish a comprehensive multimodal dataset, assess the feasibility of multimodal data collection in an acute clinical setting, and provide a foundation for subsequent methodological development, classification, and predictive modeling studies rather than to test a specific hypothesis. The sample size was therefore based on feasibility, available resources, and the expected number of eligible patients during the study period.

### Statistical Methods

We assessed normality of the continuous variables with the Shapiro-Wilk test. As age and number of diseases were not normally distributed, we used the Kruskal-Wallis test to evaluate differences between the controls, patients with TIA, and patients with stroke. To determine which groups differed statistically significantly, we conducted the Dunn post hoc test with Benjamini-Hochberg correction. To assess group differences in the categorical variables, we used the Fisher exact test. In all analyses, we set the significance level to .05. We performed the analyses using R (version 4.4.1; R Foundation for Statistical Computing), Python (version 3.8.17; Python Software Foundation), and Python packages *numpy* (version 1.24.2) [40], *pandas* (version 1.5.3) [41], *pingouin* (version 0.5.5) [42], *scipy* (version 1.10.1) [43], and *scikit-posthocs* (version 0.8.1) [44].

Given the multimodal nature of the dataset, data quality considerations differ across modalities and intended analyses. Therefore, quality control procedures, exclusion criteria, preprocessing methods, and handling of missing values will be specified separately for each analytical study. Measurements with substantial artifacts, technical failures, or incomplete recordings may be excluded from the corresponding analyses. The dataset is intentionally maintained as close to the original recordings as possible to allow different research questions and analytical approaches, including statistical and machine learning methods, to apply modality-specific data curation strategies as appropriate.

### Planned Analytical Approaches

As this paper presents the study protocol and cohort description, the statistical analyses reported here are limited to characterization of the study population and comparison of participant groups. However, the multimodal dataset was specifically designed to support subsequent data-driven analyses aimed at improving CVD diagnosis, treatment response assessment, recovery prediction, and identification of risk factors and biomarkers. In addition to descriptive and group comparison analyses, the multimodal dataset may enable the development of data-driven models for classification and prediction tasks. These may include differentiating between stroke, TIA, and healthy controls, as well as predicting clinical outcomes such as recovery based on the mRS. Feature extraction from sensor-based data, including time-series, image, and video data, can be combined with clinical and questionnaire data to develop supervised machine learning models. As this is a protocol study, specific modeling approaches and performance evaluations will be reported in subsequent studies. These may include supervised machine learning models (eg, classification and regression models), including multimodal data fusion approaches integrating physiological, clinical, and behavioral features.

## Results

This multicenter study protocol was successfully conducted at 2 Finnish university hospitals including various data collection methods. In total, we recruited 263 participants, of whom, 123 were healthy controls (43 in KUH and 80 in OUH), 31 were patients with TIA (11 in KUH and 20 in OUH), and 103 were patients with stroke (85 in KUH and 18 in OUH). We excluded 6 participants: 5 had other conditions than TIA or stroke, and 1 control had a previous CVD accident. Of the patients with stroke, 100 had ischemic stroke, and 3 had hemorrhagic stroke. Participant flow through the study is presented in Figure 3, and the availability of usable data across modalities, assessments, and follow-up measures is presented in Table 2. Differences in sample sizes reflect modality-specific eligibility criteria, participant ability to complete assessments, technical failures, incomplete assessments, and loss to follow-up. The patients with stroke and TIA were recruited from October 2021 to September 2022, and healthy controls from October 2021 until December 2022. Participant characteristics are presented in Table 3, including demographic variables, disease burden, and clinical outcomes at 3-month follow-up.

**Figure 3:**
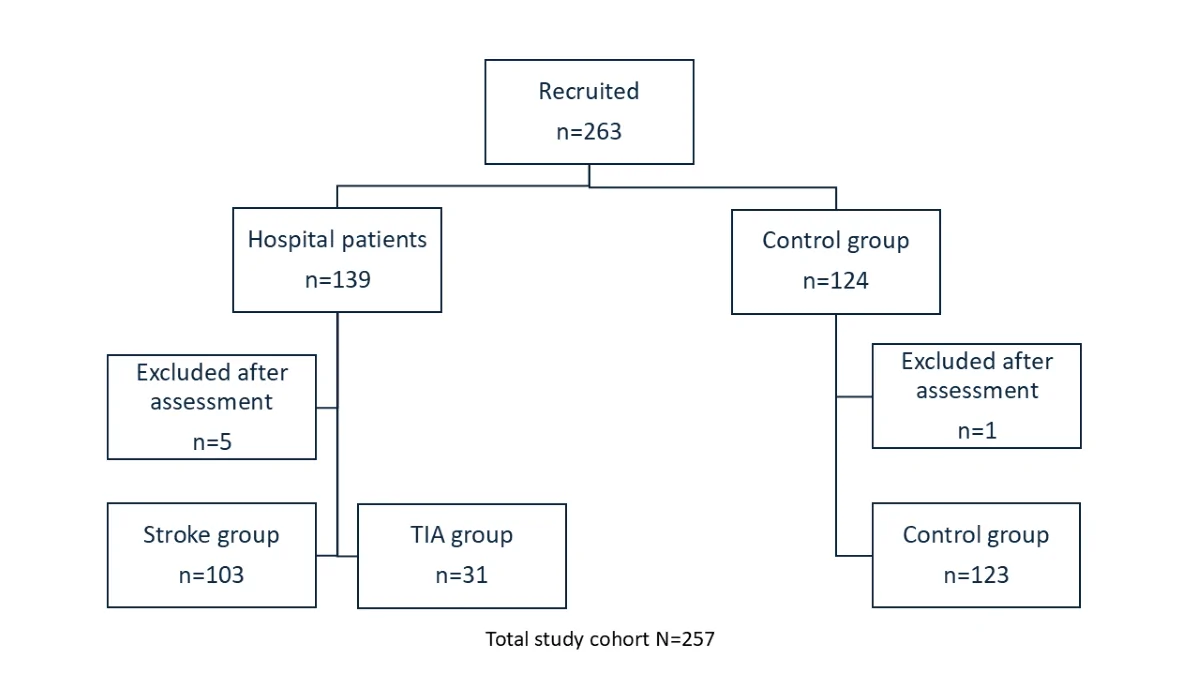
Participant flow diagram. Of the 263 recruited participants, 257 were included in the final study cohort after exclusions. In total, 5 participants were excluded after completion of the study assessments because their final diagnosis was revised to a condition other than stroke or TIA, and 1 control participant was excluded because a previous CVD was identified. Classification of participants with stroke and TIA was based on the final clinical diagnosis established during the hospital stay. TIA: transient ischemic attack.

**Table 2:** Availability of collected data across modalities and follow-up assessments.

Data type	Controls, n	TIA^a^, n	Stroke, n	Total, n
Final study cohort	123	31	103	257
Questionnaires	123	31	103	257
mRS^b^ at discharge	—^c^	31	103	134
NIHSS^d^ and NIRS^e^ and EEG^f^ and ECG^g^	117	26	82	225
NIHSS and EEG	117	28	82	227
NIHSS and video	54	10	84	148
Gait	121	29	70	220
Fundus imaging	121	26	73	220
3-Month follow-up	—	27	93	120

^a^TIA: transient ischemic attack.

^b^mRS: modified Rankin Scale.

^c^Not available.

^d^NIHSS: National Institutes of Health Stroke Scale.

^e^NIRS: near-infrared spectroscopy.

^f^EEG: electroencephalography.

^g^ECG: electrocardiography.

**Table 3:** Characteristics of study participants (healthy controls, patients with transient ischemic attack [TIA], and patients with stroke) in a prospective multicenter study conducted at 2 Finnish university hospitals between October 2021 and December 2022^a^.

Variable			Control (n=123)		TIA (n=31)			Stroke (n=103)		*P* value	
Age (years), median (IQR)			59 (45-72)		71 (60-80)			70 (59-76)		K-S^b^: <.001^c^	
**Sex, n (%)**									Missing: n=1		
	Female	84 (68.3)		8 (25.8)			42 (41.2)			<.001	
**Marital status, n (%)**									Missing: n=1		.29
	Married or registered partnership	64 (52)		17 (54.8)			54 (52.9)				
	Cohabiting	21 (17.1)		1 (3.2)			20 (19.6)				
	Divorced or widow	26 (21.1)		9 (29)			22 (21.6)				
	Single	12 (9.8)		4 (12.9)			6 (5.9)				
**Education, n (%)**									Missing: n=1		<.001
	Primary education^d^	28 (22.8)		13 (41.9)			32 (31.4)				
	Upper secondary education	34 (27.6)		11 (35.5)			47 (46.1)				
	Bachelor or equivalent	38 (30.9)		3 (9.7)			12 (11.8)				
	Master, doctoral, or equivalent	23 (18.7)		4 (12.9)			11 (10.8)				
**Work situation, n (%)**			Missing: n=1					Missing: n=1		<.001	
	Employee	64 (52.5)		9 (29)			25 (24.5)				
	Entrepreneur	3 (2.5)		2 (6.5)			10 (9.8)				
	Retired	53 (43.4)		20 (64.5)			66 (64.7)				
	Student	1 (0.8)		0 (0)			0 (0)				
	Parental leave or stay-at-home parent	1 (0.8)		0 (0)			0 (0)				
	Unable to work or on sick leave	0 (0)		0 (0)			1 (1)				
**Number of previous cerebrovascular accidents, n (%)**									Missing: n=3		<.001
	0	122 (99.2)		20 (64.5)			79 (79)				
	1	1 (0.8)		6 (19.4)			16 (16)				
	2-3	0 (0)		5 (16.1)			5 (5)				
**Number of diseases**									Missing: n=3		
	Median (IQR)	1 (0-2)		2 (1-3.5)			2 (1-2)			K-S: <.001^e^	
**Modified Rankin Scale at 3 months, n (%)**						Missing: n=4			Missing: n=10		<.001
	0=no symptoms at all	N/A^f^		20 (74.1)			22 (23.7)				
	1=no significant disability despite symptoms	N/A		7 (25.9)			36 (38.7)				
	2=slight disability	N/A		0 (0)			30 (32.3)				
	3=moderate disability	N/A		0 (0)			4 (4.3)				
	4=moderately severe disability	N/A		0 (0)			0 (0)				
	5=severe disability	N/A		0 (0)			0 (0)				
	6=dead	N/A		0 (0)			1 (1.1)				

^a^There were no missing values, unless otherwise stated.

^b^K-S: *P* value from Kruskal-Wallis test.

^c^*P* value for control versus TIA comparison and control versus stroke comparison is <.001; *P* value for TIA versus stroke comparison is .41.

^d^International Standard Classification of Education≤2.

^e^*P* value for control versus TIA comparison and control versus stroke comparison is <.001; *P* value for TIA versus stroke comparison is .57.

^f^N/A: not applicable.

## Discussion

### Principal Findings

This study describes the design and implementation of a comprehensive multimodal data collection protocol aimed at supporting improved diagnosis, outcome prediction, and biomarker identification in CVDs.

Cerebrovascular accidents are critical neurological emergencies that require immediate diagnosis and treatment since they may have devastating consequences on the quality of life of the patients. Furthermore, accurate differential diagnostics between stroke and TIA is important so that the appropriate treatments for these different accidents can be made efficiently. Therefore, novel methods for faster and more accurate diagnosis of patients with CVD are needed.

This study describes the design and cohort characteristics of the Stroke-Data study, which aimed to address the need for prompt diagnosis by investigating the physiological markers from patients with CVD and controls comprehensively and combining information collected from different sources in a new way. More specifically, we successfully collected EEG, ECG, NIRS, gait, fundus imaging, and video recordings of neurological examination and combined them with clinical assessments, questionnaires, and EHR data.

### Comparison With Prior Work

The strength of the study is its uniqueness, since, to our knowledge, there are no other studies that have applied this large number of different modality measurements to the same participants. Studies in the literature collected only 1 modality for stroke detection, such as EEG signals [45-47], fundus imaging [48-50], and neurological examination video analysis [28,51]. Another strength of the study is that it included both patients with stroke and TIA, in addition to healthy controls.

### Strengths and Limitations

The study has several limitations. First, the number of participants was relatively low, as collecting comprehensive multimodal data from patients undergoing a life-threatening condition requiring immediate diagnosis and care is challenging. Second, the study does not include longitudinal follow-up of healthy participants and therefore does not enable prediction of first-ever stroke or TIA events or prospective risk modeling. Third, there were differences in characteristics between healthy controls and disease groups due to the challenges in recruiting comparable participants. Although the original aim was to recruit healthy controls with a similar age and sex distribution as the patient groups, exact matching was not achieved because of recruitment challenges. Specifically, the healthy controls included a higher proportion of female participants and were more highly educated, generally younger, and more often employed than participants in the disease groups. These differences may introduce confounding effects when comparing groups and may influence observed associations in both statistical analyses and data-driven models. Consequently, the findings should be interpreted with caution, particularly when generalizing results or developing predictive models based on group comparisons. In addition, the relatively small size of certain subgroups, such as patients with TIA and hemorrhagic stroke cases, as well as incomplete data across some modalities, may limit the development and generalizability of predictive models and should be addressed in future larger-scale studies. Future studies should aim to include better-matched control groups or apply statistical approaches to adjust for these differences.

Some of the technologies used in this study, such as EEG and NIRS, are resource-intensive and require trained personnel, which may limit their direct applicability in routine clinical practice, particularly in acute stroke settings. These modalities were included to provide comprehensive physiological information and to support methodological development. Future research should evaluate which subset of modalities provides sufficient diagnostic or predictive accuracy while remaining feasible for implementation in clinical environments.

### Future Directions

Although initial findings from the dataset have already been published, this protocol paper is the first to provide a comprehensive description of the study design and multimodal data collection framework. This documentation is important for transparency, reproducibility, and interpretation of both existing and future studies using the dataset.

To conclude, we successfully collected multimodal data from healthy controls and patients with TIA and stroke, and the data can be used for studying stroke- and TIA-related biomarkers, disease progression, recovery, and differences between patient groups and healthy controls, as well as for creating novel methods that support early and accurate diagnosis of CVDs. With these multimodal data, data-driven prediction models can be developed, which aim to identify the patients or groups of patients who are most likely to benefit from certain treatment or rehabilitation. Simultaneously, data sources and features can be identified to predict the patient’s recovery. This enables health care resources and procedures to be targeted more precisely, so that the treatment result benefits the patient, and the resulting treatment and rehabilitation costs can be optimized at the societal level.

## Appendix

Multimedia Appendix 1 Detailed data collection protocol.

## Abbreviations

ABC: Activities-Specific Balance Confidence
ABCD^2^: a risk stratification score based on age, blood pressure, clinical features, duration, and diabetes
CT: computed tomography
CVD: cerebrovascular disease
ECG: electrocardiography
EEG: electroencephalography
EHR: electronic health record
fNIRS: functional near-infrared spectroscopy
ICD: International Classification of Diseases
KUH: Kuopio University Hospital
mRS: modified Rankin Scale
NIHSS: National Institutes of Health Stroke Scale
NIRS: near-infrared spectroscopy
OUH: Oulu University Hospital
TIA: transient ischemic attack
