# Supplementary material for: Novel Sensors and Data-Driven Approach to Support Prevention, Diagnosis, and Treatment Planning of Cerebrovascular Accidents: Protocol for a Multimodal Data Collection Study With Novel Sensors
Source: JMIR Res Protoc. 2026 Oct 8;15:e86930. doi: 10.2196/86930 (PMC13691472; doi:10.2196/86930)
Supplement: Supplementary file 1 [file resprot_v15i1e86930_app1.docx]

### Video and sound

The video data collection protocol was designed to reflect standard clinical neurological examination procedures, focusing on tasks included in the National Institutes of Health Stroke Scale (NIHSS). The study nurses took an online course for obtaining certificates for National Institutes of Health Stroke Scale (NIHSS) protocol to be able to score the test steps according to the NIHSS-protocol.

A custom-made smartphone application was developed for the video data collection to support the study protocol and to ensure information security and privacy of the study subjects. Video and sound data collection is performed only with the subject’s consent. The Nokia X20 smart phone model was selected for the video data collection due to the camera quality and operating system. The phone was not connected to mobile network or Wi-Fi-network during the data collection.

Video data collection protocol:

1. The study nurse explains the test protocol for the subject.
2. The study nurse enters the subject’s study ID into the mobile application.
3. The study nurse follows the instructions by the application and starts the recording.
4. The subject is asked to perform the following tasks:
a. The subject is asked to smile, show their teeth, and raise their eyebrows for a few times.
b. The subject is asked to follow an object with their eyes by holding their head in the forward position. The object is moved from left to right.
c. The subject is asked to read aloud sentences presented on paper.
d. The subject is asked to explain what happens in a picture shown to them (picture used in NIHSS protocol).
e. The subject is first asked to rise one arm straight forward with palm down either in supine or sitting position (in supine position to 45° angle and in sitting position to 90° angle) with eyes closed and hold for ten seconds. The same task is repeated with the other arm.
f. The subject is asked in supine position to rise one leg to 30° angle and hold for five seconds. The same task is repeated with the other leg.
g. If the subject can stand, the subject is asked to stand still for 30 seconds.

The smartphone application shows timestamps for the recording process for study nurses to follow as a guide while performing the NIHSS steps. In addition, the results of the NIHSS tests are saved in the subject's data folder with a recording tool.

### EEG, NIRS, Video and sound

The video data collection protocol was repeated with EEG and NIRS measurements. The same protocol was followed to be able to collect annotation information while measuring EEG and NIRS signals. The video data collection application included timestamps of each phase, and the patient data collection software included scoring of each phase of NIHSS-protocol.

EEG, ECG, NIRS, Video, and sound -protocol:

1. The study nurse presents the test protocol for the subject.
2. The study nurse enters the subject’s study ID to the measurement application on a laptop computer and to the mobile application for video data collection.
3. The study nurse puts the sensor cap on the subject's head. The sensor cap includes DC-EEG, NIRS and accelerometer sensors.
4. The study nurse attaches the ECG sensor patch to the chest of the subject.
5. The study nurse starts the recording of DC-EEG, NIRS, movement sensor and ECG on the laptop computer and video data collection on the mobile device according to instructions provided to the study nurse.
6. The subject is asked to lie still for five minutes and then sit still for five minutes to record baseline signals.
7. The subject is asked to breathe and hold breath for a moment to calibrate CBF reserve (Cerebral Blood Flow).
8. The same protocol as in the video data collection protocol is performed (see video data collection protocol 3. & 4. a-g.)
9. After the tasks are finished, the recording is stopped on the laptop computer and mobile device.
10. The study nurse detaches the sensors from the subject and performs the appropriate ECG sensor patch disposals and cleans the cap.

### Accelerometer data collection from gait

The subject’s dynamic balance and walking ability was studied with a gait test. The test was done only for those patients that were able to walk independently a distance of 10 meters. A 3D acceleration sensor was attached to the patient’s lower back for the gait test, and the measurement data were transferred wirelessly to a mobile device. An easy-to-use mobile application developed for this purpose (Similä et al. 2018) was used for the data collection. This application is used to start and stop the measurement. The mobile application stored the acceleration signal collected from the subject’s gait combined with the study ID. The mobile phone was not connected to mobile network or Wi-Fi-network during the data collection, but the Bluetooth-connection was used for the data transfer between the sensor and the mobile phone.

Gait study protocol:

1. The study nurse assesses the subject's ability to walk independently for approximately 10 meters. If the subject can perform the test, the study nurse explains the test protocol and purpose of the measurement to the subject.
2. The study nurse attaches the accelerometer on the subject's waist with the included fastening belt in a way that the sensor is located to the lower back of the study subject.
3. The study nurse enters the subject's predetermined study ID into the application.
4. The study nurse follows the instructions provided by the mobile application; check that there is enough space for the gait test, for example more than 10 meters long corridor. When the subject is ready, the study nurse starts measurement from the "Start" button of the application and stops the measurement from the "Stop" button. If needed, the measurement can be repeated.

It takes approximately 5 minutes to complete the gait study protocol. The mobile application is used only by the study nurse, and the subject is not given any feedback about their gait.

### Fundus images

Optomed Aurora Camera was used to take photos of the retinas of both eyes of the subject, i.e. the fundus. The images were first saved on the camera's memory card, from which they were transferred for later use to the disk space used by the study. Description was not required, for example the use of pupil-dilating subjects or other preliminary preparations. The shooting event corresponded to normal shooting with a digital camera. The harm caused to the patient was limited to the use of flashlight to the resulting momentary glare. Fundus study protocol was as follows:

1. The study nurse introduces the test protocol to the subject.
2. The study nurse describes the subject's fundus according to the given instructions (training, user manual, other educational material).
3. At the end of the recording, the study nurse transfers the images from the camera to a separately defined storage space.

The estimated duration of the measurement protocol for each subject is 5 minutes. In addition, it takes approximately one minute for the study nurse to clean the device. Two pictures from both eyes were taken. In the first image the central visual field is in the middle of the image and in the second image the papilla is in the middle. The device’s user interface instructs the user to take the pictures in correct order. After taking the picture, the image appears on the display. If the quality of the image is sufficient, it is accepted by the study nurse, and the next picture is taken. If the quality of the picture is not good enough, a new picture should be taken. Sometimes it is impossible to get a successful image even by trying several times. In this study, the image is tried three times before moving to the next imaging phase.

### Questionnaire: Lifestyle-related cerebrovascular risk factors

These questions were translated from Finnish.

*When answering the following questions, think about a month preceding the cerebrovascular accident, due to which you are now in the hospital.*

1. Do you smoke?
   a. No, I do not smoke at all
   b. I quit in year ___________
   c. I smoke an average ___________ cigarettes per day and I have smoked since ___________
   d. I use other tobacco products, what? ___________
2. Have you felt stressed or depressed?
   a. Yes, very much
   b. Yes, somewhat
   c. No, not at all
3. How much exercise did you get daily (e.g., by bike/walk, chores at home, walking to the shop, physical exercise, etc.)?
   a. Less than 1 hour per day
   b. 1-2 hours a day
   c. More than 2 hours a day
4. How often did you do light physical activity in your free time? Light physical activity = do not get out of breath or sweat
   a. Once a month or less often
   b. 2-3 times a month
   c. Once a week
   d. 2-3 times a week
   e. 4-6 times a week
   f. Daily
5. How often did you do moderate-intensity physical activity in your free time? Moderate physical activity = you get out of breath and sweat at least slightly
   a. Once a month or less
   b. 2-3 times a month
   c. Once a week
   d. 2-3 times a week
   e. 4-6 times a week
   f. Daily
6. Did you even occasionally consume any alcoholic beverages? E.g. beer, cider, mild wines, wine or spirits?
   a. I do not use
   b. I use, on average, __________ servings per week. One serving (= 12 g) is a bottle of medium strength beer or cider; a glass of mild wine; a small glass of fortified wine; restaurant portion of spirits.
7. Do you think you got enough good quality sleep on average?
   a. Yes
   b. Somewhat adequately
   c. No, I do not
8. Do you follow a special diet? E.g. lactose-free, gluten-free, vegetarian diet.
   a. Yes, what? ________________________________________
   b. I do not
9. On average, how many meals did you eat daily? Meals include e.g. breakfast, lunch, snack, dinner, evening snack. _______________________________

### Questionnaire: Patient experience

These questions were translated from Finnish.

With the help of the following statements, we collect feedback about the care you received. When answering, think about the entire care path from the onset of the symptoms until now. Choose the option that suits you best.

1. The doctors spoke to me in an understandable way.
2. The nurses spoke to me in an understandable way.
3. The personnel were skilled and professional.
4. The personnel were interested in my situation.
5. The personnel treated me kindly.
6. I received good and appropriate treatment.
7. I was taken care of comprehensively.
8. I felt that the pain was adequately treated.
9. I received enough information about my treatment.
10. My family members/those close to me received enough information about my treatment.
11. Decisions about my treatment were made in cooperation with me.
12. I found the service I received useful.
13. My privacy was respected.
14. The facilities were functional and comfortable.
15. The operation of the hospital was well organized.
16. According to my own assessment, there were no mistakes in my treatment.
17. Overall assessment of the service: the service was so good that I can recommend it.

Answer options: strongly disagree, somewhat disagree, neither agree nor disagree, somewhat agree, strongly agree.

18. Open question:

What other feedback would you like to give regarding your care experience? Please note that sensitive information, such as personal identification numbers or information about your health, should not be included in the feedback. In matters related to your actual treatment, please contact the hospital personnel directly.
